# Gastric Outlet Obstruction as a Result of an Inguinal Hernia

**DOI:** 10.5811/cpcem.35395

**Published:** 2025-01-19

**Authors:** Luke Wohlford, Richard Bounds

**Affiliations:** The University of Vermont Medical Center, Department of Emergency Medicine, Burlington, Vermont

**Keywords:** gastric outlet obstruction, inguinal hernia

## Abstract

**Case Presentation:**

We present a case of a 79-year-old male with gastric outlet obstruction resulting from a stomach herniation through a large left inguinal hernia.

**Discussion:**

Stomach-containing inguinal hernias are a rare cause of gastric outlet obstruction. Treatment options range from conservative to surgical management. Once identified with imaging, prompt treatment should be initiated to prevent incarceration, strangulation, and gastric necrosis.

## CASE PRESENTATION

A 79-year-old male with a left inguinoscrotal hernia and aortic stenosis presented to the emergency department with an acute syncopal episode. The patient attributed his own weakness and passing out to poor oral intake and abdominal pain for three days. He noted a left inguinal bulge that had never bothered him previously. The gastrointestinal symptoms had improved the day prior to the patient’s syncopal episode, but he started to feel palpitations and generally weaker on the day of presentation. He had no prior surgeries for the inguinal hernia, which had no previous complications.

**Image 1:**
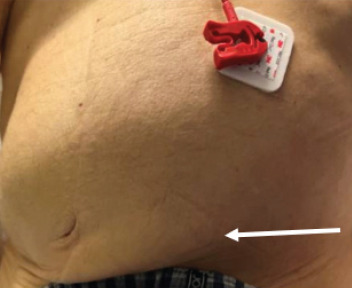
Physical examination demonstrating a grossly distended and palpable left inguinal mass (white arrow)

**Image 2:**
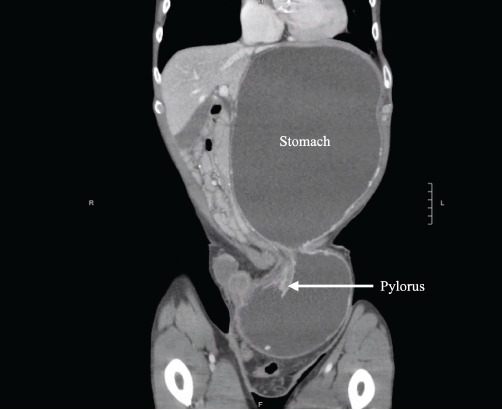
Computed tomography with coronal images demonstrating a stomach-containing inguinal hernia including the pylorus.

**Image 3:**
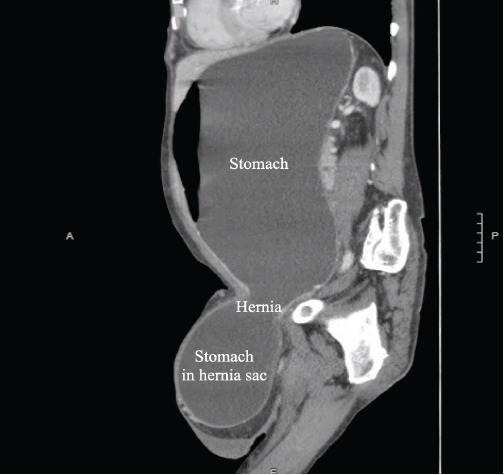
Computed tomography with sagittal images demonstrating a large and distended stomach partially in an inguinal hernia.

On examination, vital signs included blood pressure of 123/82 millimeters of mercury, pulse rate 141 and irregularly irregular, respiratory rate 16 breaths per minute, and temperature 36.8° Celsius. The abdominal exam demonstrated a distended but non-tender abdomen, with a palpable left inguinal mass (Image 1). Further workup with computed tomography demonstrated a large and distended stomach with extension of the distal portion including the pylorus into the inguinal hernia (Images 2 and 3).

## DISCUSSION

Incarceration of a portion of the stomach is a rare cause of gastric outlet obstruction, with fewer than 20 cases documented in the literature.^1^ The rarity of stomach-containing groin hernias is remarkable, considering that the lifetime incidence of groin hernias is estimated to be between 27–43% for men and 3–6% for women.^2^ The inferior portion of the stomach attaches to the omentum by the gastrocolic ligament, making it vulnerable to herniation through particularly large and chronic inguinal hernias.^3^ Acquired inguinal hernias are typically direct, resulting from the chronic pressure of a hernia sac just medial to the inferior epigastric vessels.^4^ Less common than direct hernias, but seen in our patient, is the acquired, indirect hernia protruding lateral to the inferior epigastric vessels, which travels through the inguinal canal and often extends into the scrotum. Nasogastric tube placement may adequately relieve the most severe symptoms, which can be followed by operative hernia repair, or even percutaneous endoscopic gastronomy tube placement.^5^ Physicians should be aware of the ability of the stomach to herniate into large inguinal hernia defects and act promptly to avoid incarceration, strangulation, and stomach necrosis.

**CPC-EM Capsule**

What do we already know about this clinical entity?

*Stomach-containing inguinal hernias are rare and can potentially lead to gastric outlet obstruction if left untreated*.

What is the major impact of the image(s)?

*The computed tomography images depict herniation of the stomach through the inguinal canal, including the gastric antrum, demonstrating a unique cause of gastric outlet obstruction*.

How might this improve emergency medicine practice?

*This case highlights the importance of thorough physical examination and consideration of imaging to prevent serious, hernia-related complications*.
